# Acceleration of osteoblast differentiation by a novel osteogenic compound, DMP-PYT, through activation of both the BMP and Wnt pathways

**DOI:** 10.1038/s41598-017-08190-9

**Published:** 2017-08-16

**Authors:** Su Jung Bae, Hye Joo Kim, Hee Yeon Won, Yong Ki Min, Eun Sook Hwang

**Affiliations:** 1 Center for Drug Discovery Technology, Korea Research Institute of Chemical Technology, Daejeon, 34114 Korea; 2 College of Pharmacy and Graduate School of Pharmaceutical Sciences, Ewha Womans University, Seoul, 03760 Korea; 3 Immunotherapy Convergence Research Center, Korea Research Institute of Bioscience and Biotechnology, Daejeon, 34141 Korea

## Abstract

Osteoblast differentiation is regulated through the successive activation of signaling molecules by a complex interplay of extracellular signals such as bone morphogenetic protein (BMP) and Wnt ligands. Numerous studies have identified natural as well as synthetic compounds with osteogenic activity through the regulation of either BMP/SMADs or the Wnt/β-catenin pathway. Here, we attempted to isolate small molecules that concurrently activated both SMADs and β-catenin, which led to the discovery of a novel potent osteogenic compound, DMP-PYT. Upon BMP2 stimulation, DMP-PYT substantially increased osteoblast differentiation featured by enhanced expression of osteoblast-specific genes and accelerated calcification through activation of BMPs expression. DMP-PYT promoted BMP2-induced SMAD1/5/8 phosphorylation and β-catenin expression, the latter in a BMP2-independent manner. DMP-PYT alone enhanced nuclear localization of β-catenin to promote the DNA-binding and transcriptional activity of T-cell factor, thereby resulting in increased osteoblast differentiation in the absence of BMP2. Most importantly, DMP-PYT advanced skeletal development and bone calcification in zebrafish larvae. Conclusively, DMP-PYT strongly stimulated osteoblast differentiation and bone formation *in vitro* and *in vivo* by potentiating BMP2-induced activation of SMADs and β-catenin. These results suggest that DMP-PYT may have beneficial effects for preventing and for treating osteoporosis.

## Introduction

Bone tissue is composed of bone-forming osteoblasts and bone-resorbing osteoclasts. Bone mass is homeostatically regulated by the interplay of osteoblasts and osteoclasts^1, 2^. Osteoblasts express receptor activator of NF-κB ligand (RANKL), which binds to its receptor, RANK, on the extracellular surface of osteoclasts^3, 4^. RANKL–RANK interaction stimulates osteoclast differentiation and bone resorption activity, whereas osteoprotegerin, produced by osteoblastic stromal cells, interrupts this interaction through competitive binding to RANKL, thus inhibiting osteoclast differentiation^5^. Additionally, osteoclasts regulate osteoblast differentiation and bone-forming activity by producing cytokines, such as transforming growth factor-beta (TGFβ)^6^. Although it is argued that TGFβ is critically required for osteoclast differentiation, TGFβ and bone morphogenetic proteins (BMPs) that belong to the TGFβ superfamily stimulate osteoblast differentiation through activation of the TGFβ receptor or BMP receptor signaling pathways^7^. Imbalance between bone formation and resorption causes various bone disorders, including osteoporosis and osteopetrosis.

Osteoblasts are differentiated from bone marrow mesenchymal stem cells upon stimulation with extracellular signals that activate intracellular signaling molecules. In particular, extracellular BMPs bind to their receptors and activate receptor kinases, resulting in the phosphorylation of specific SMAD proteins. Activated SMADs translocate into the nucleus to increase the transcription of genes encoding osteoblast-specific factors, such as runt-related transcription factor 2 (RUNX2), osteocalcin, matrix extracellular phosphoglycoprotein, and alkaline phosphatase^8^. In addition, BMP signaling activates β-catenin, a Wnt signal transducer, to induce osteoblast differentiation^9^. Upon BMP stimulation, β-catenin accumulates and locates to the nucleus. Nuclear β-catenin interacts with T-cell factor/lymphoid enhancer-binding factor (TCF/LEF) proteins to promote TCF/LEF-mediated gene transcription^10^. BMP and Wnt cooperatively activate SMAD- and β-catenin-mediated osteoblast gene expression and accelerate osteoblast differentiation, implicating the importance of the BMP and Wnt signaling pathways in bone formation^7, 11^.

Various studies have attempted to isolate small molecules that activate the BMP and Wnt signaling pathways. Dorsomorphin derivatives and flavonoids were identified as BMP inhibitors or activators^12–15^, and several compounds were newly characterized to down- or up-regulate Wnt/β-catenin signaling^16, 17^. However, these compounds mainly control the ligand-receptor binding complex or receptor-associated membrane proteins as agonists or antagonists. It would be useful to isolate cell-permeable small compounds that can directly modulate BMP- and Wnt-mediated signaling molecules. To this end, we screened a drugable chemical library and investigated the bioactive compounds that activated both BMP/SMADs and β-catenin. We identified a novel compound, DMP-PYT, which strongly promoted bone formation *in vitro* as well as *in vivo* through phosphorylation of BMP/SMADs and nuclear accumulation of β-catenin.

## Results

### Screening for osteogenic compounds that activate the BMP2/SMADs and β-catenin

**Figure 1:**
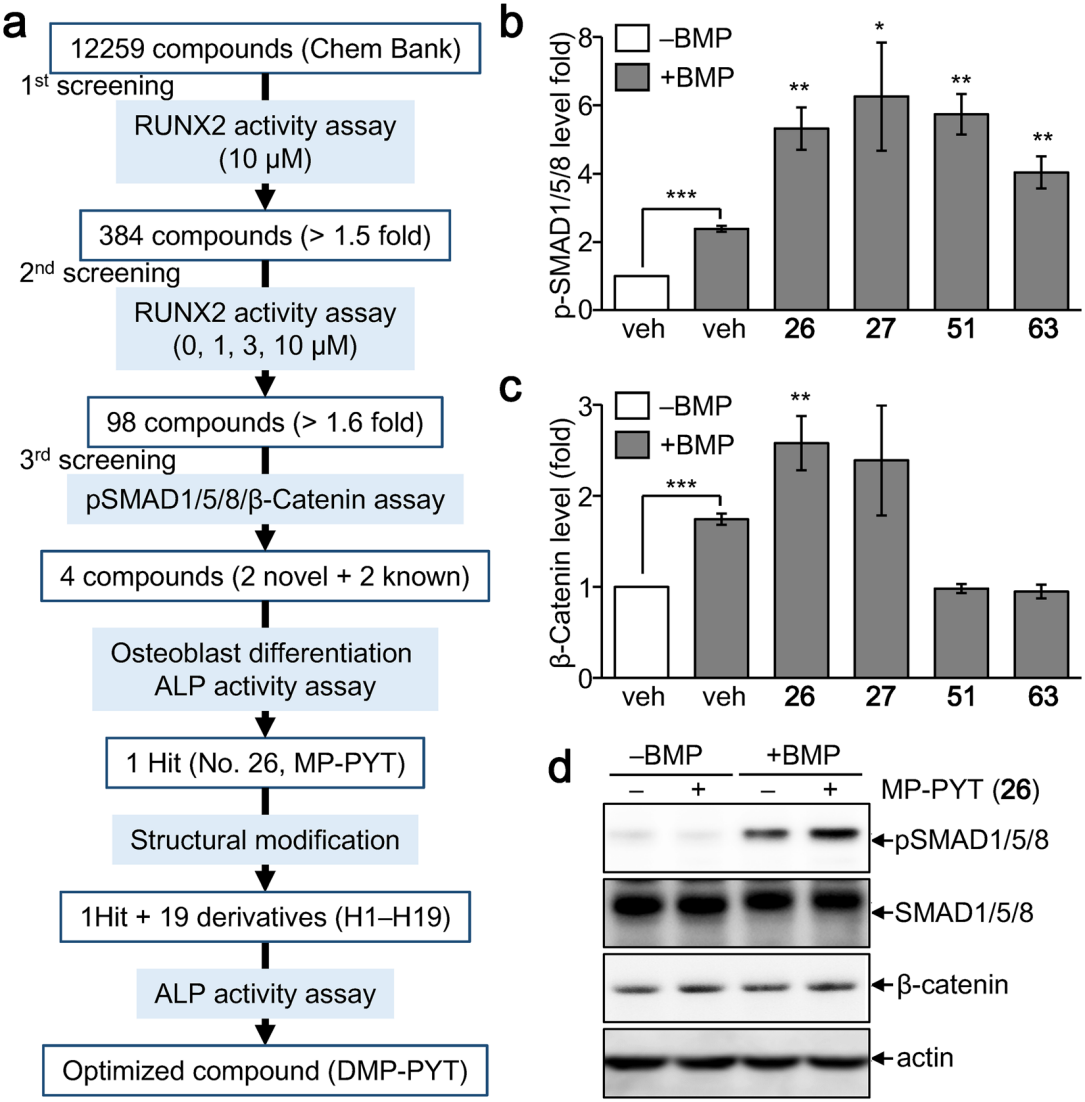
Isolation of novel osteogenic compounds. (**a**) A selection cascade used for isolation of osteogenic compounds from the chemical library (Korea Chemical Bank, http://www.chembank.org). Bioactive compounds were narrowed down through the sequential selection from RUNX2 activity assay, SMAD phosphorylation (pSMADs) assay, β-catenin expression assay, ALP activity assay, and ALP staining after osteoblast differentiation. (**b**,**c**) Confluent C2C12 cells were treated with a 10 µM of the selected compounds (**26**, **27**, **51**, and **63**) in the presence of BMP2 (25 ng/ml) for 30 min and subjected to immunoblotting of pSMADs and β-catenin, followed by protein extraction. Quantitation of pSMADs (**b**) and β-catenin (**c**) band intensity was determined from three independent experiments by densitometry using Image J software. The averages of expression level of pSMADs (**b**) and β-catenin (**c**) are shown. *P < 0.05, **P < 0.005, and ***P < 0.0005. (**d**) Confluent C2C12 cells were treated with MP-PYT (**26**) in the absence or presence of BMP2 (25 ng/ml) for 30 min and collected for immunoblotting analysis with antibodies against pSMAD1/5/8, SMAD1/5/8, β-catenin, and β-actin.

We attempted to isolate potent osteogenic compounds that boost SMAD phosphorylation and β-catenin activation in response to BMP2 stimulation through sequential selection (Fig. 1a). High-throughput and subsequent dose-dependent reporter assays using a chemical compound library narrowed down the number of bioactive compounds to 98 (Supplementary Fig. S1). Immunoblotting and quantitative analysis revealed that four compounds significantly increased the phosphorylation of SMADs (Supplementary Fig. S2 and Fig. 1b). The effects of these four compounds on β-catenin expression and alkaline phosphatase (ALP) activity were comparatively analyzed. All four compounds substantially enhanced ALP activity (Supplementary Fig. S3). However, compound **26**, 5-(3-(4-(dimethylamino)phenyl)allylidene)-1-(3,5-methylphenyl)pyrimidine-2,4,6 (1H, 3H, 5H)-trione (MP-PYT), more potently increased BMP2-induced β-catenin expression than the others (Fig. 1c). Additional immunoblotting confirmed that MP-PYT increased the expression of pSMAD1/5/8 synergistically in the presence of BMP2 and also moderately induced β-catenin expression (Fig. 1d).

### Identification of DMP-PYT with a strong osteogenic activity

**Figure 2:**
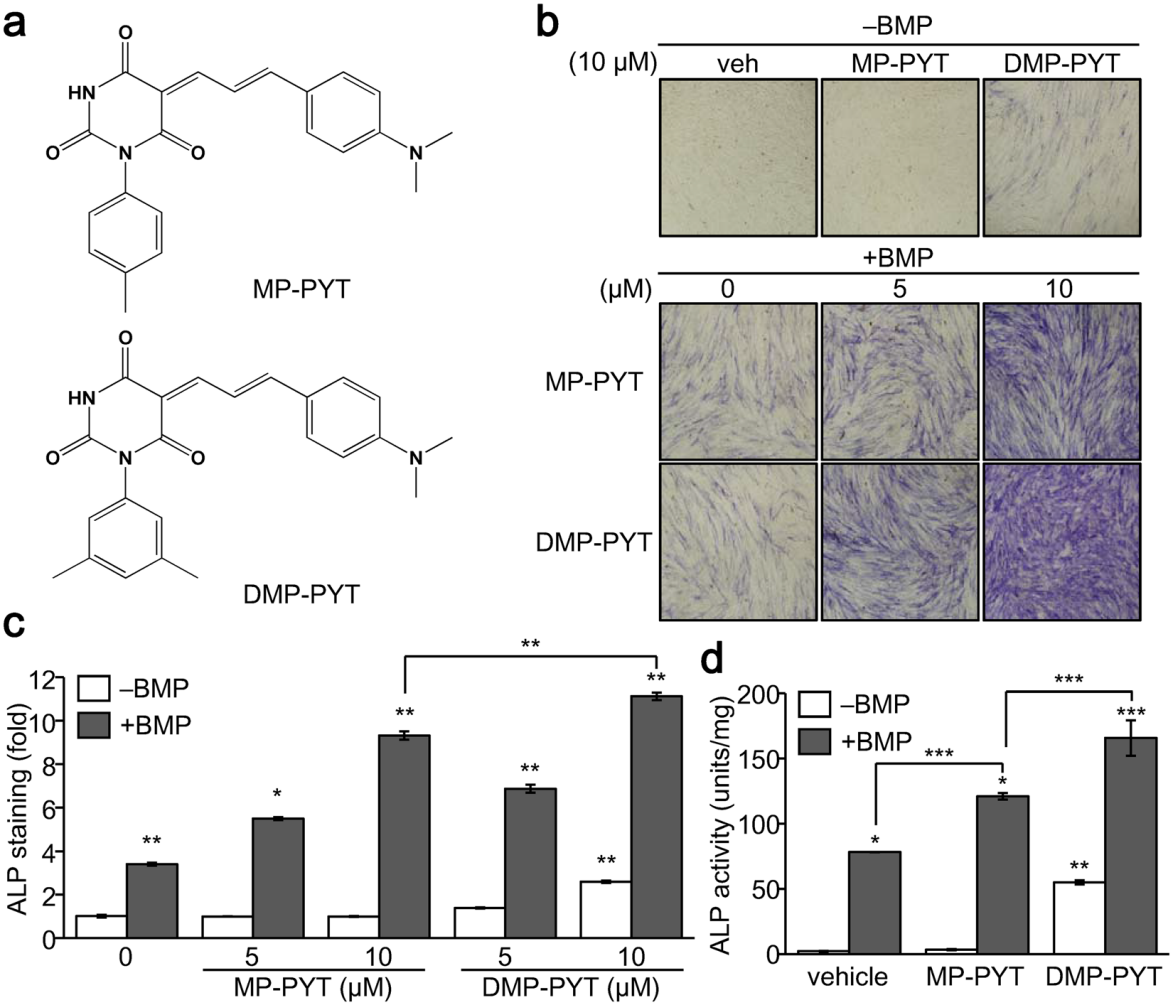
Isolation of novel osteogenic compounds. (**a**) Structure of MP-PYT and DMP-PYT. (**b–d**) C2C12 cells were induced to differentiate into osteoblast in the presence of BMP2 (25 ng/ml) and treated with MP-PYT (5 and 10 µM) and DMP-PYT (5 and 10 µM) for 6 days. Cells were fixed and incubated with ALP staining solution for 30 min. Cell images were then captured at 10x magnification using a microscope (Olympus IX51) (**b**). Ten microscopic cell images were acquired and processed with the Image J Cell counter analysis tool. ALP staining intensity is expressed as a fold change relative to the control (−BMP) (**c**). Cell extracts were harvested and used for ALP activity assay (**d**). At least three independent experiments were performed. *P < 0.05, **P < 0.005, and ***P < 0.0005.

As MP-PYT showed osteogenic potential, we asked whether structural changes of MP-PYT would promote the osteogenic property. Thus, we compared the osteogenic activity of 19 additional compounds that are structurally similar to MP-PYT (Supplementary Fig. S4). Interestingly, compound H19, 5-(3-(4-(dimethylamino)phenyl)allylidene)-1-(3,5-dimethyl-phenyl)pyrimidine-2,4,6 (1H, 3H, 5H)-trione (DMP-PYT), more potently increased ALP activity than did MP-PYT and the other compounds (Supplementary Fig. S5). DMP-PYT shares a dimethylphenyl allylidene pyrimidine trione moiety with MP-PYT, but contains a dimethyl phenyl moiety instead of a methyl phenyl group (Fig. 2a). BMP-induced osteoblast differentiation of C2C12 cells was increased by MP-PYT, and more so by DMP-PYT, as evidenced by ALP staining and quantitative analysis (Fig. 2b,c). Accordingly, MP-PYT up-regulated ALP activity in BMP2-induced osteoblast differentiation, while DMP-PYT significantly and more potently elevated this activity in a dose-dependent manner (Fig. 2d). While DMP-PYT and BMP2 synergistically enhanced osteogenic differentiation, DMP-PYT significantly stimulated osteogenic differentiation even in the absence of BMP2 (Fig. 2b–d). There were no significant effects of DMP-PYT on cell viability and proliferation of osteoblast lineage cells (Supplementary Fig. S6).

### Enhanced transcription of osteoblast-specific markers by DMP-PYT treatment

**Figure 3:**
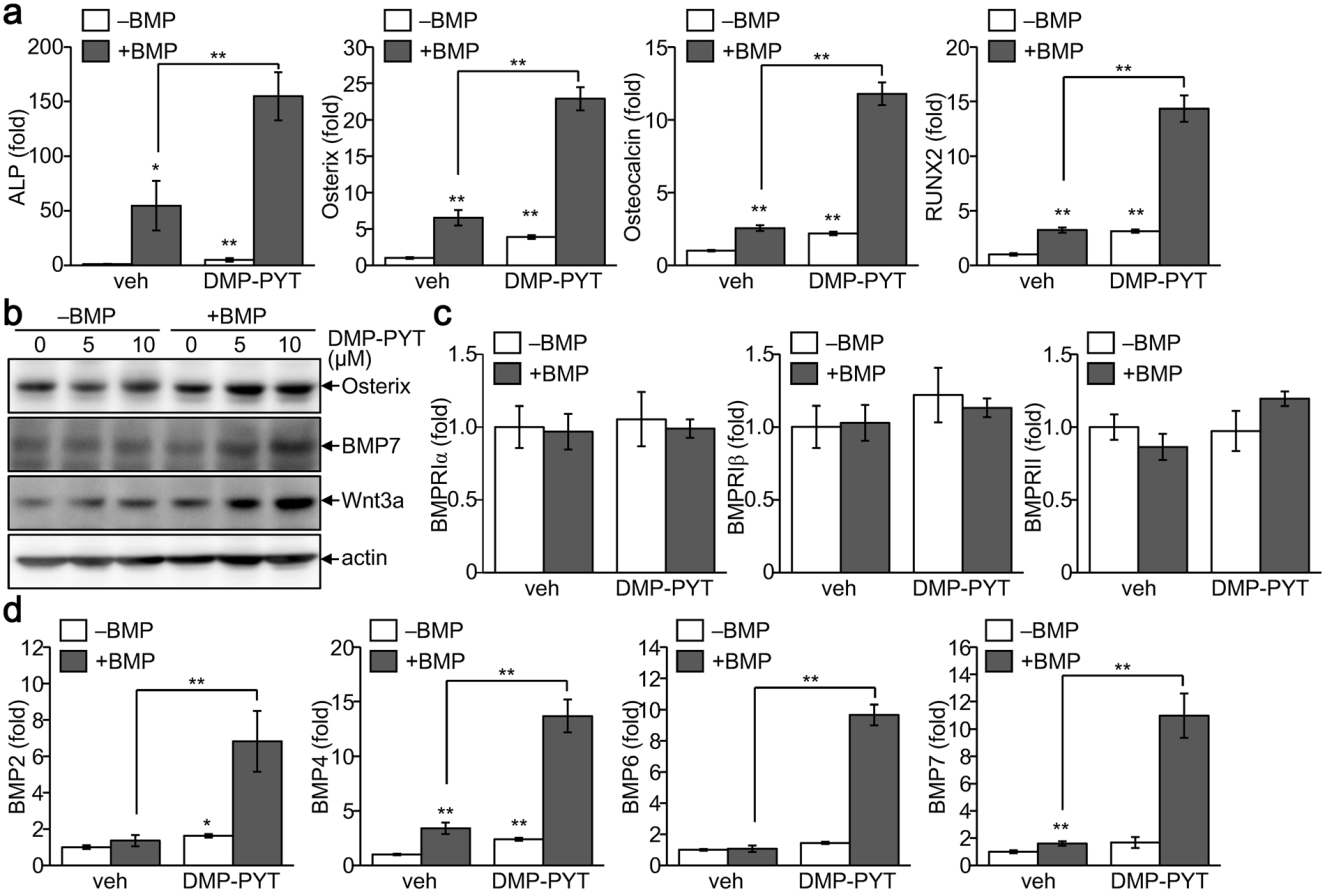
Increased expression of osteoblast-specific markers by DMP-PYT. C2C12 cells were grown to confluence and treated with DMP-PYT in the absence (−BMP) or presence (+BMP) of BMP2. Cells were refreshed every 3 days and differentiated into osteoblasts for 6 days. (**a**) Total RNA was prepared from differentiated cells and subjected to reverse transcription and real-time PCR analysis. Relative transcript levels of ALP, osterix, osteocalcin, and RUNX2 were determined after normalization with GAPDH level. (**b**) Total protein lysates were harvested and analyzed by immunoblotting using antibodies against osterix, BMP7, WNT3a, and β-actin. (**c**,**d**) Relative mRNA levels of BMPR molecules BMPR1α, 1β and II (**c**), and BMP molecules BMP2, BMP4, BMP6, and BMP7 (**d**) were quantitatively determined by quantitative real-time PCR analysis and is expressed as fold changes after normalization to GAPDH level. *P < 0.05 and **P < 0.005.

As DMP-PYT potently enhanced osteoblast differentiation and calcium deposition during differentiation, we next analyzed the expression levels of the osteoblast-specific markers ALP, osterix, osteocalcin, and RUNX2. Quantitative real-time PCR analysis demonstrated that BMP2 significantly up-regulated osteoblast marker expression, and DMP-PYT together with BMP2 more potently enhanced the transcripts of osteoblast-specific markers (Fig. 3a). Consistently, the protein level of osterix was increased by BMP and DMP-PYT (Fig. 3b). Interestingly, BMP7 and Wnt3a, signaling molecules that potently activate and promote osteoblast differentiation, were also increased by DMP-PYT with BMP2 (Fig. 3b). We further examined whether DMP-PYT increased expression or activity of BMPs and BMP receptors (BMPR). There was no significant change in the expression levels and kinase activity of BMPR1β and BMPRII after treatment with DMP-PYT (Fig. 3c and Supplementary Fig. S7a). However, the expression of BMP2, BMP4, BMP6, and BMP7 was significantly up-regulated at the transcriptional level by DMP-PYT in the presence of BMP2 stimulation, whereas non-osteogenic BMP3 and activin expression was not influenced by DMP-PYT (Fig. 3d and Supplementary Fig. S7b).

### BMP-dependent SMAD activation and BMP-independent β-catenin induction by DMP-PYT

**Figure 4:**
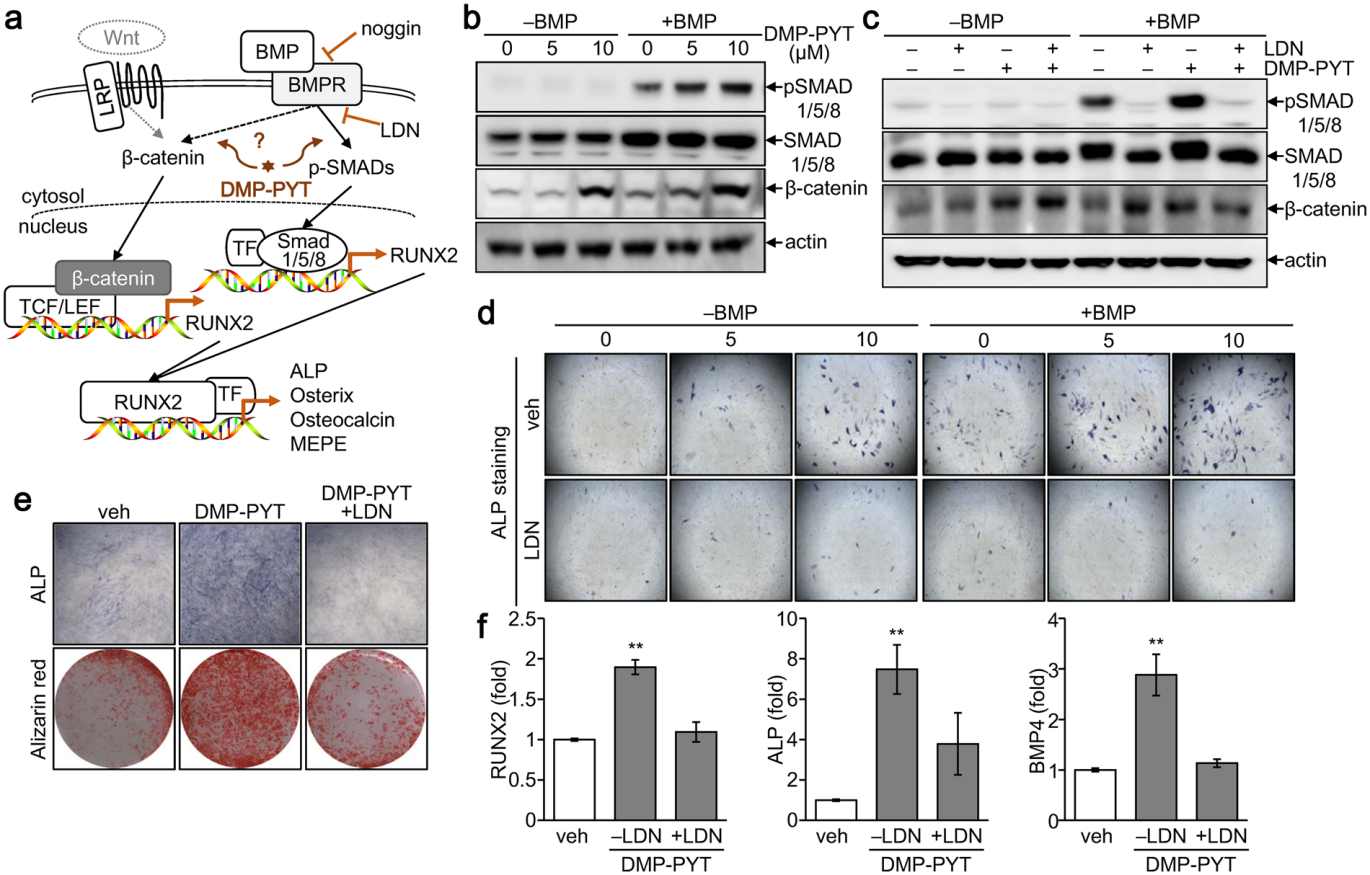
Enhanced BMP signaling by DMP-PYT during osteoblast differentiation. (**a**) A schematic diagram showing BMP-mediated signaling cascade. (**b**) C2C12 cells were plated and treated with DMP-PYT (5 and 10 µM) for 30 min in the absence or presence of BMP2 (25 ng/ml). Total protein lysates were harvested and analyzed by SDS-PAGE and immunoblotting with antibodies against pSMAD, SMAD, β-catenin, and β-actin. (**c**) Separately, cells were incubated with DMP-PYT (10 µM) for 30 min in the absence or presence of the BMP inhibitor, LDN-193189 (100 nM) and BMP2 (25 ng/ml). Total protein lysates were freshly harvested and assayed for immunoblot analysis with antibodies for pSMAD, SMAD, β-catenin, and β-actin. (**d**) C2C12 cells were incubated with DMP-PYT (10 µM) and/or LDN-193189 (100 nM) in the absence or presence of BMP2 (25 ng/ml). Cells were then stained with ALP staining solution, followed by the microscopic observation. (**e**,**f**) MC3T3-E1 cells were incubated with DMP-PYT (10 µM) and/or LDN-193189 (100 nM) and induced to differentiate into osteoblast. Cells were then stained with ALP and Alizarin red S, followed by microscopic observation (**e**). Total RNA was collected and reversely transcribed into cDNA for quantitative real-time PCR analysis of RUNX2, ALP, BMP4, and GAPDH (**f**). **P < 0.005.

As DMP-PYT stimulated BMP2-induced osteoblast differentiation, we next investigated the underlying molecular mechanisms. BMP stimulation immediately induces the phosphorylation of SMAD1/5/8 and also increases β-catenin activity by protein stabilization^18^, which then transcriptionally activates RUNX2 expression and subsequently RUNX2-mediated osteoblast gene expression (Fig. 4a). We asked whether BMP2 stimulation was prerequisite for DMP-PYT function in activation of SMAD1/5/8 and β-catenin. We observed that phosphorylated SMAD1/5/8 were induced by BMP2 and further increased by treatment with DMP-PYT (Fig. 4b) and that the increased phosphorylation of SMAD1/5/8 by DMP-PYT was suppressed by the presence of LDN-193189, a BMP2 inhibitor (Fig. 4c). Consistently, osteogenic activity of DMP-PYT was significantly suppressed by the inhibition of BMPR, not TGFβR, signaling (Fig. 4d, Supplementary Fig. S9a). Moreover, DMP-PYT significantly enhanced osteogenic differentiation of MC3T3-E1 cells without BMP2 stimulation, which was also inhibited by LDN-193189 (Supplementary Fig. S9b, Fig. 4e). The increased expression of osteogenic markers after treatment with DMP-PYT was accordingly decreased by the presence LDN-193189 (Fig. 4f). We additionally observed that DMP-PYT had no significant effects on non-canonical BMP signaling molecules but substantially increased phosphorylated p38 independent of BMP2 stimulation. Furthermore, activation of pSMAD1/5/8 by DMP-PYT was also enhanced by TGFβ treatment, whereas TGFβ-induced activation of pSMAD2/3 was rather suppressed by DMP-PYT (Supplementary Fig. S8).

### Enhanced nuclear localization and DNA-binding activity of β-catenin by DMP-PYT

**Figure 5:**
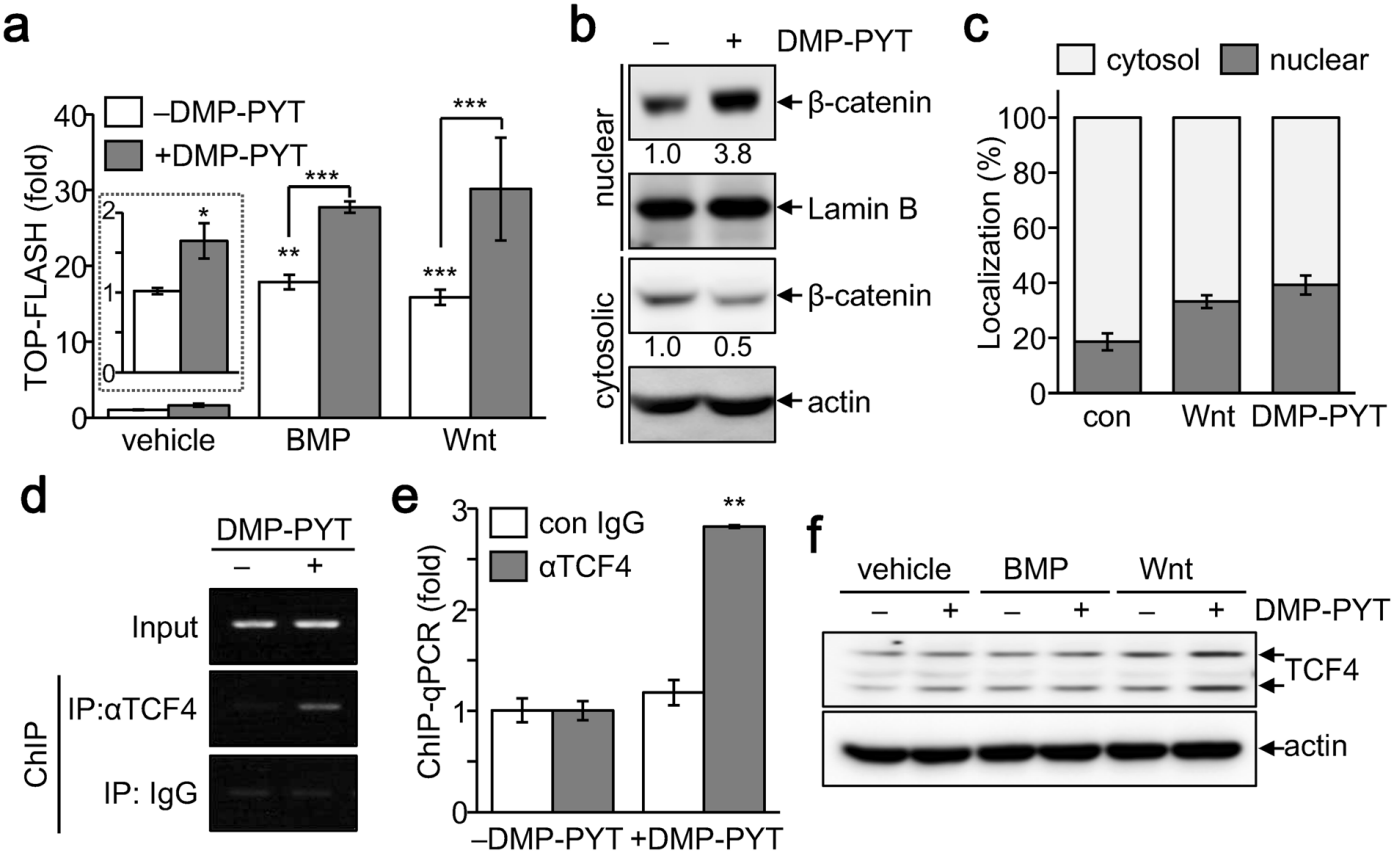
Enhanced nuclear localization and DNA-binding activity of β-catenin by DMP-PYT. (**a**) C2C12 cells were transfected with reporter genes TOP-FLASH and pCMVβ using calcium phosphate transfection method and then incubated with DMP-PYT (10 µM) together with either BMP (25 ng/ml) or Wnt3a (50 ng/ml) for an additional 24 h. Relative reporter activity was determined after normalization with β-galactosidase activity and expressed as fold induction. (**b**) MC3T3-E1 cells were treated with DMP-PYT (10 µM) for 4 h and harvested for fractionation of nuclear and cytosolic proteins. Proteins were resolved by SDS-PAGE and incubated with antibodies against β-catenin, Lamin B, and β-actin for immunoblotting analysis. (**c**) Cells were treated with DMP-PYT (10 µM), fixed, and stained with β-catenin antibody, followed by confocal microscopic observation. Nuclear and cytosolic localization was determined by quantitative analysis of the images using Image J program. (**d**,**e**) MC3T3-E1 cells were treated with DMP-PYT (10 µM) for 48 h under osteoblast differentiation conditions and harvested for chromatin immunoprecipitation assay. Total lysates were incubated with anti-TCF4 antibody and immune complexes were subjected to a semi-quantitative PCR (**d**) and real-time PCR analysis (**e**) for TCF4-binding element within BMP2 promoter. **P < 0.005. (**f**) Total protein was analyzed by immunoblotting with anti-TCF4 antibody after treatment with DMP-PYT (10 µM) together with either BMP2 (25 ng/ml) or Wnt3a (50 ng/ml).

As DMP-PYT substantially increased the expression of β-catenin and osteoblast-specific gene transcription without addition of exogenous BMP, we assessed its effect on β-catenin signaling. We found that TOP-FLASH reporter activity that is induced by β-catenin-activated TCF/LEF, was elevated upon stimulation with BMP2 or Wnt3a, and was further increased by the treatment with DMP-PYT (Fig. 5a). In addition, DMP-PYT moderately but significantly increased the reporter activity in the absence of BMP2 and Wnt3a (Fig. 5a). We thus asked whether DMP-PYT affected the subcellular localization of β-catenin. Interestingly, DMP-PYT treatment increased β-catenin expression particularly in the nuclear fraction, while cytoplasmic β-catenin was attenuated by DMP-PYT (Fig. 5b). Quantitative analysis of these results demonstrated that DMP-PYT enhanced nuclear localization of β-catenin as much as Wnt stimulation did (Fig. 5c). Furthermore, TCF protein, which associates with β-catenin for DNA-binding, was observed to strongly bind to the chromatin DNA in the presence of DMP-PYT, as evidenced by chromatin-immunoprecipitation (ChIP) assay and quantitative real-time PCR (Fig. 5d,e). However, DMP-PYT had no significant effect on TCF protein expression (Fig. 5f).

### Increased bone formation by DMP-PYT *in vitro* and *in vivo*

**Figure 6:**
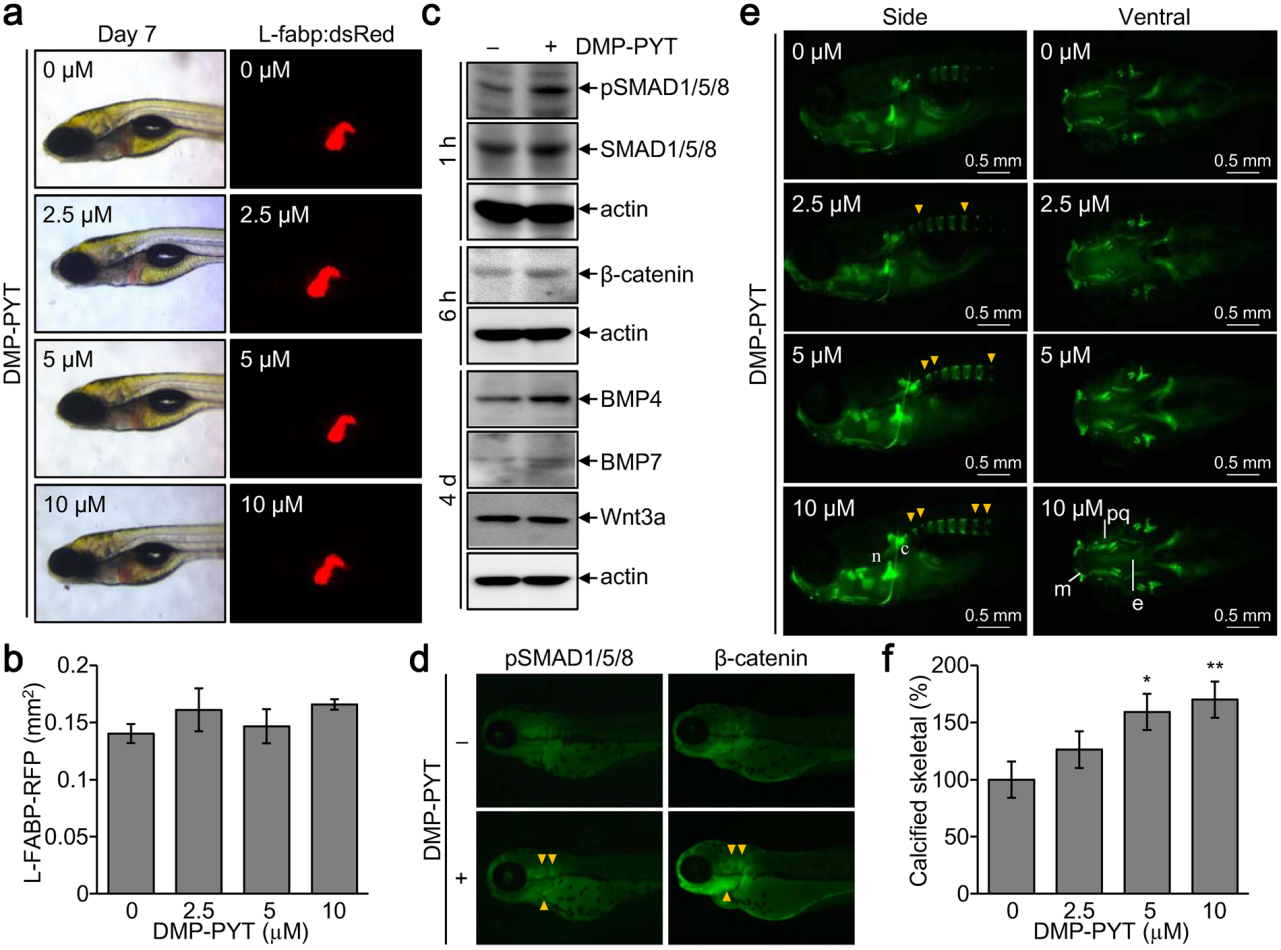
Increased calcified skeletal development by DMP-PYT *in vivo*. L-Fabp:dsRed zebrafish embryos (n = 10) were incubated with the indicated amounts of DMP-PYT for 6–7 days post fertilization. (**a**) Zebrafish were observed under a fluorescence stereomicroscope and ref fluorescence was observed in the liver. (**b**) The hepatic size showing red fluorescence was quantitatively determined from 10 larvae images by automatically calculating the fluorescent area (mm^2^) in the liver. (**c**,**d**) Zebrafish larvae (n = 10) at day 3 post fertilization were treated with DMP-PYT for 1 h, 6 h, and 4 days and harvested for immunoblotting analysis. Protein blots were incubated with antibodies against pSMAD1/5/8, SMAD1/5/8, β-catenin, BMP4, BMP7, Wnt3a, β-actin, followed by enhanced chemiluminescence and autoradiography (**c**). Fish larvae that were treated with DMP-PYT for 1 and 6 h and subjected to immunohistochemistry of pSMAD1/5/8 and β-catenin (**d**). (**e**) Skeletal development of zebrafish was visualized by calcein staining at day 7 post fertilization. Side view of the head skeleton: n, notochord; c, cleitrum. Ventral view of the head skeleton: m, Meckel’s cartilage; pq, palate quadrate; e, ethmoid plate. Arrowheads indicate the increased bone formation in vertebrate skeleton. (**f**) Calcification of axial skeleton was quantitatively determined from 10 larvae images using image J software and expressed as the percentage of the control. *P < 0.05 and **P < 0.005.

Next, we aimed to confirm the osteogenic potential and activation of BMP and Wnt signaling molecules induced by DMP-PYT *in vivo* and evaluated whether DMP-PYT affected skeletal bone formation in zebrafish larvae development. First, *in vivo* hepatic cytotoxicity of DMP-PYT was assayed in Tg(zf.L-fabp:DsRed) zebrafish, which selectively expresses red fluorescence in the liver during larval development^19^. Administration of DMP-PYT did not affect hepatic red fluorescence in the zebrafish larvae (Fig. 6a,b). We then examined signaling molecules related to BMP2 and Wnt pathways in zebrafish larvae after treatment with DMP-PYT. DMP-PYT treatment increased the phosphorylation of SMAD1/5/8 and β-catenin at 1 h and 6 h, respectively, and elevated the expression of BMP4 and BMP7 at later stage of larvae development (Fig. 6c). Enhanced expression of pSMAD1/5/8 and β-catenin by DMP-PYT was also ascertained by immunohistochemistry analysis (Fig. 6d). We finally examined the effects of DMP-PYT on zebrafish skeletal development by calcein staining at day 7 post fertilization. DMP-PYT administration dose-dependently increased ossification in the zebrafish larvae, most notably in the axial skeleton (Fig. 6e,f).

## Discussion

In an attempt to isolate potent osteogenic compounds, we found that the pyrimidine trione derivatives MP-PYT and DMP-PYT strongly increased osteoblast differentiation by activating SMADs and β-catenin in the presence of BMP2. Comparative analysis confirmed that DMP-PYT showed significantly stronger effects than MP-PYT in promoting BMP2-induced osteoblast differentiation. Furthermore, DMP-PYT significantly enhanced osteoblast-specific gene expression by promoting BMP2-induced SMAD signaling. Interestingly, DMP-PYT increased the expression of β-catenin regardless of the presence of BMP2, and it enhanced the nuclear localization of β-catenin and promoted DNA-binding of TCF/β-catenin, thereby enhancing the expression of Wnt/β-catenin target genes. Most importantly, DMP-PYT boosted skeletal development in zebrafish larvae *in vivo*, suggesting its beneficial effects for preventing or treating osteoporosis.

Although we clearly demonstrated that DMP-PYT enhanced osteoblast differentiation, the underlying molecular mechanisms remain largely elusive. DMP-PYT induced the activation of SMADs and β-catenin as well as the expression of the osteoblast-specific transcription factor RUNX2, which in turn led to elevated expression of the osteoblast markers RUNX2, ALP, osteocalcin, and osterix. Phosphorylation of SMAD1/5/8 induced by DMP-PYT was partially dependent of BMP2 because DMP-PYT also upregulated the expression of TGFβ-induced pSMAD1/5/8, not pSMAD2/3. In the absence of BMP2 stimulation, DMP-PYT facilitated the phosphorylation of SMAD1/5/8 and increased the expression of BMPs including BMP2 and BMP4. These results suggest that DMP-PYT may induce the autoactivation of BMPR signaling through induction of osteogenic BMPs. Exogenous BMP2 addition fortified the expression of BMPs that were directly or indirectly induced by DMP-PYT. As a result, DMP-PYT-induced osteogenic activity was completely diminished by blockade of BMP2 signaling but was not affected by TGFβ receptor inhibitors. However, promotion of β-catenin activation by DMP-PYT was BMP2-independent. It will be interesting to uncover whether DMP-PYT affects BMP2 receptor kinase, glycogen synthase kinase-3, or phosphatase activity to increase SMAD phosphorylation, and how DMP-PYT regulates β-catenin expression and nuclear localization in a BMP2-independent manner. As DMP-PYT is suggested to control both the BMP2/SMAD and β-catenin signaling pathways, it will also be important to identify which signaling pathway plays a key role in DMP-PYT-induced osteoblast differentiation. In addition to the bone-forming effects of DMP-PYT in embryos, it will be necessary to demonstrate whether DMP-PYT can prevent or cure osteoporosis in mammals *in vivo*.

Although numerous types of small molecules have been identified to treat osteoporosis^20^, most marketed drugs decrease bone loss by inhibiting bone resorption^21^. In particular, bisphosphonates are widely used representative anti-osteoporotic drugs that mainly suppress bone resorption and enhance bone formation^22^. Although bisphosphonates have powerful dual abilities to control both bone resorption and formation, increased fracture risk has been reported in clinic as an adverse effect^23^. More novel anti-osteoporotic agents targeting cathepsin K and c-Src kinase are under development for clinical use^24^. Recent studies report that immune cells such as T helper 17 and regulatory T cells are involved in the bone destruction of osteoporosis, and immune modulators have been suggested as potential anti-osteoporotic drug candidates^25, 26^. However, upcoming therapies will be replaced with bone anabolic drugs that can increase bone mass by directly enhancing osteoblast differentiation and bone-forming activity^27^. The bone anabolic potential of various proteins, including insulin growth factor-1, parathyroid hormone-related proteins, dickkopf-related protein-1, sclerostin, and Wnt signaling molecules, has been extensively characterized and these molecules have been proposed as therapeutic targets for treating osteoporosis^28^. A recombinant form of parathyroid hormone was developed and approved as a bone anabolic drug^29^. However, no anabolic small-molecule anti-osteoporotic drugs are available. Although DMP-PYT has a long way to go in drug development, our findings highlight its potential as an anabolic anti-osteoporotic drug candidate.

## Materials and Methods

### Materials

Chemical library compounds were obtained from Korea Chemical Bank (KRICT, Daejeon, Korea), chemical derivatives (H1 through H19) were purchased from InterBioScreen (Moscow, Russia). The structure and stereochemistry of all compounds including DMP-PYT (InterBioScreen) were confirmed by various physicochemical analytical methods and provided by KRICT and InterBioScreen with 91– 98% purity and endotoxin-free. Recombinant human BMP2, ascorbic acid, β-glycerophosphate, and ALP staining kit were purchased from R&D (Minneapolis, MN) and Fluka (Basel, CH, Germany), and Sigma-Aldrich Corp. (St. Louis, MO).

### Cell culture and osteoblast differentiation

Mouse cell lines C2C12 (CRL-1772, ATCC, Manassas, VA) and MC3T3-E1 subclone 4 (CRL-2593, ATCC) were maintained in DMEM containing 10% heat inactivated FBS (Thermo Fisher Scientific, Waltham, MA). C2C12 cells (10^5^ cells/ml) were grown to confluence for 48 h and replaced with DMEM containing BMP-2 (25 ng/ml, R&D Systems, Minneapolis, MN) for inducing osteoblast differentiation. Cells were replaced with fresh DMEM containing BMP2 at day 3 and cultured for 6 days. MC3T3-E1 cells (10^5^ cells/ml) were cultured and grown to confluence in αMEM supplemented with 10% FBS, glutamine (2 mM), and penicillin and streptomycin (100 U/ml). Cells were then replaced with complete αMEM containing ascorbic acid (50 µg/ml) and β-glycerophosphate (10 mM) for osteoblast differentiation. Cells were refreshed every 3 days and induced to differentiate into osteoblasts for 10 days.

### Alkaline phosphatase (ALP) & alizarin red S staining and ALP activity assay

For ALP staining, differentiated osteoblasts were washed with PBS and fixed in 10% formalin for 2 min. Cells were rinsed with deionized water and incubated with alkaline dye mixture for 30 min by wrapping with aluminum foil according to the manufacturer’s instruction (Sigma-Aldrich). Cells were then rinsed with deionized water and subjected to a microscopic observation. For alizarin red S staining, cells were washed with distilled water, fixed in ice-cold 70% ethanol for 1 h, and then stained with 2% Alizarin red S stain solution (adjusted to pH 4.2 with 10% ammonium hydroxide) (Sigma-Aldrich) for 30 min. Stained cells were washed and observed using a light microscope. At least 10 cell images were captured under a microscope (Olympus IX51 inverted fluorescent microscope, Olympus Optical, Japan) fitted with a DP70 camera system (Olympus) and used for a quantitative analysis with the Image J software. To determine ALP activity, cells were washed and lysed with Triton X-100 lysis buffer (50 mM Tris, 150 mM NaCl, and 1% Triton X-100, pH 10). Cell supernatants were incubated with an ALP substrate. ALP activity assay was performed in triplicate using the LabAssay ALP Kit (Wako Pure Chemical Industries, Japan). Relative ALP activity was calculated by comparing with standard solution and expressed as units per mg of protein according to the manufacturer’s instructions.

### Immunoblotting analysis

Total cell lysates were obtained by lysis in RIPA lysis buffer with protease inhibitors (Roche, Germany). Nuclear and cytosolic proteins were fractionated using NE-PER Nuclear and Cytoplasmic Extraction kit (Thermo Fisher Scientific) and protein concentration was determined by bicinchoninic acid protein assay kit (Thermo Fisher Scientific). Proteins (20 µg) were resolved by SDS-PAGE and transferred to an Immobilon-P membrane (Millipore, Bedford, MA). Protein blots were incubated with antibodies against pSMAD1/5/8 (Cell signaling, Beverly, MA), β-catenin (Millipore), RUNX2 (Abcam, Cambridge, MA), SMAD1/5/8, BMP4, BMP7, Wnt3a, Lamin B, and β-actin (Santa Cruz Biotechnology Inc., Santa Cruz, CA). Specific protein bands were detected by chemiluminescence imaging with LAS-3000 (Fujifilm, Japan).

### Revers transcription and real time PCR analysis

Total RNA was isolated using a TRIzol reagent (Invitrogen, Carlsbad, CA) and reversely transcribed using a cDNA synthesis kit (QIAGEN Korea Ltd, Seoul, Korea). The cDNA was then used as template for real-time PCR using SYBR Green pre-mix buffer (Applied Biosystems, Foster City, CA) and a specific primer set. Primers were as follows: 5′-gccgggaatgatgagaacta-3′, 5′-ggaccgtccactgtcacttt-3′ for RUNX2; 5′-gggacccgctgtcttctagt-3′, 5′-tcaactcaaattcgctgagga-3′ for BMP2; 5′-cctggtaaccgaatgctgat-3′, 5′-agccggtaaagatccctcat-3′ for BMP4; 5′-caacgccctgtccaatgac-3′, 5′-actcttgcggttcaaggagtg-3′ for BMP6; 5′-cgataccaccatcgggagttc-3′, 5′-aaggtctcgttgtcaaatcgc-3′ for BMP7; 5′-tgctgtattgctgacctggg-3′, 5′-gttccagcggttagacacga-3′ for BMPR1α; 5′-atgcctgttgtcacctctgg-3′, 5′-acttcccgagctctgagact-3′ for BMPR1β; 5′-agcacagaggcccaattctc-3′, 5′-ttagacactgtggtcgtggc-3′ for BMPRII; 5′-atgggcgtctccacagtaac-3′; 5′-tcacccgagtggtagtcaca-3′ for ALP; and 5′- aactttggcattgtggaagg-3′, 5′-acacattgggggtaggaaca-3′ for GAPDH. Real-time PCR was performed using an ABI-Prism 7700 sequence detector (Applied Biosystems) and the relative expression level was calculated after normalization to the level of GAPDH.

### Reporter gene assay

C2C12 cells were transfected with TOP-FLASH, a reporter gene containing the β-catenin/TCF binding element linked to the luciferase gene by liposome-mediated transfection (Invitrogen) and then treated with DMP-PYT compound for an additional 24 h. A pCMVβ vector was used as an internal control to compensate for the transfection efficiency. Cell extracts were harvested in a reporter lysis buffer. Reporter activity was assayed with a luciferase assay kit (Promega, Madison, WI) or a β-galactosidase assay kit (Thermo Fisher Scientific). Relative luciferase activity was determined after normalization to the β-galactosidase activity and expressed as a fold induction.

### Immunofluorescence staining and quantitative analysis

MC3T3-E1 cells were treated with DMP-PYT and fixed in formalin. Cells were then permeabilized with 0.1% Triton X-100 in PBS and subsequently incubated with antibodies against active β-catenin (Millipore). Cells were incubated with secondary antibody conjugated with Alexa Fluor 488 (Molecular Probes, Invitrogen). Nuclei were counterstained using Hoechst 33342 (Life Technologies, Rockville, MD). Cells that express β-catenin in the nucleus were counted from 10 different images of three independent experiments. Results are mean ± SE and expressed as percentage compared to the control group. Nuclear localization of β-catenin was determined in Wnt3a-treated cells as a positive control.

### Chromatin immunoprecipitation (ChIP) assay

ChIP assays were performed using MAGnify™ ChIP according to the manufacturer’s instructions (Thermo Fisher Scientific). In brief, C2C12 cells were incubated with DMP-PYT for 24 h and fixed in formaldehyde for 10 min. Cell suspensions were sonicated using High power sonication under the optimized condition (20 cycle; 30 pulses of 30 s each with a 30-s rest on ice between pulses). Cell lysates were incubated with anti-TCF4 antibody and chromatin-immune complexes were captured to magnetic beads and eluted in PBS after washing. Quantitative real-time PCR was performed using primer sets detecting TCF4 binding elements in distal promoter of BMP2 gene. The following primers were used for ChIP-PCR: 5′-gacctctacagctctagaaacag-3′ and 5′-cattcaggagccttcatagtacc-3′.

### Bone formation in zebrafish larvae development

WT and Tg (L-fabp:dsRed) zebrafish were maintained at 28 °C with a photoperiod of 14:10 h (light:dark), in 10 L glass tanks with freshwater and aeration. Spawning of zebrafish was carried out by pairing one male with two females and fish eggs were incubated in egg water (Sigma Aldrich). Adult zebrafish experiments were conducted in accordance with the specific guidelines approved by the Institutional Animal Care and Use Committee of Chungnam National University (CNU-00866). At 72 h post fertilization, the zebrafish larvae were exposed to DMP-PYT for 4 days in KRICT. At day 7 post fertilization, Tg (L-fabp:dsRed) zebrafish were then euthanized and mounted on glass slides, followed by microscopic observation using a stereomicroscope (MZ10F, Leica, Germany). L-FABP-RFP was captured and quantitatively determined from 10 larvae images by calculating the fluorescent area of the liver (Leica Application Suite software). WT zebrafish larvae were incubated with DMP-PYT for an additional 4 days at 72 h post fertilization and immersed by deionized water containing 0.2% calcein (Sigma Aldrich) for 20 min for determining bone formation. Zebrafish larvae were then mounted on glass slides and observed under a fluorescent microscope. Calcification of axial skeletal was determined by measuring fluorescence intensity from 10 larvae images in each group using the Image J software and expressed as the percentage of the control.

### Statistical analysis

All experiments were performed at least three times and data are expressed as mean ± SE. A statistical significance was determined by the one-way analysis of variance. P value under 0.05 (P < 0.05) was considered statistically significant.

## Conclusion

In conclusion, we screened chemical compounds for osteogenic activity as evidenced by increases in SMAD phosphorylation and β-catenin expression. A novel pyrimidine trione derivative, DMP-PYT, was characterized to enhance BMP-induced osteoblast differentiation through an induction of BMP-dependent phosphorylation of SMADs and BMP-independent activation of β-catenin. DMP-PYT increased the nuclear expression of β-catenin and DNA-binding activity of TCF/LEF, thereby upregulating osteoblast-specific markers. DMP-PYT significantly promoted calcium deposition and bone formation *in vitro* and *in vivo*, suggesting its beneficial effect in treating bone defects such as osteoporosis.

## Electronic supplementary material

Supplementary Info
