# Supplementary material for: Acceleration of osteoblast differentiation by a novel osteogenic compound, DMP-PYT, through activation of both the BMP and Wnt pathways
Source: Sci Rep. 2017 Aug 16;7:8455. doi: 10.1038/s41598-017-08190-9 (PMC5559513; doi:10.1038/s41598-017-08190-9)
Supplement: Supplementary file 1 — Supplementary Info [file 41598_2017_8190_MOESM1_ESM.pdf]

Acceleration of osteoblast differentiation by a novel osteogenic compound, DMP-PYT, through activation of both the BMP and Wnt pathways

Su Jung Bae[1,2,3], Hye Joo Kim[1], Hee Yeon Won[2], Yong Ki Min[1,3,*], and Eun Sook Hwang[2,*]

[1]Laboratory of chemical genomics, Drug Discovery Technology Center, Korea Research Institute of Chemical Technology, Daejeon 34114 Korea

[2]College of Pharmacy and Graduate School of Pharmaceutical Sciences, Ewha Womans University, Seoul 03760, Korea

[3]Immunotherapy Convergence Research Center, Korea Research Institute of Bioscience and Biotechnology, Daejeon 34141 Korea

Supplementary Materials and Methods

High throughput reporter activity assay

C2C12 cells were transduced with 6xOSE2-luc that can evaluate cellular RUNX2 activity[1] and plated onto Greiner LUMITRAC 96-well plates (Sigma Aldrich). Cells were incubated with 10 μM chemical compounds for an additional 24 h. Cell extracts were prepared in a reporter lysis buffer and subjected to detection of luminescence using EnVision Multilabel Reader (Perkin Elmer, Boston, MA). Fold induction of reporter activity was determined after calculation was compared with the activity in Vehicle-treated cells.

Revers transcription and real time PCR analysis

Total RNA was prepared for reverse transcription and real time PCR analysis. Relative expression was determined after normalization to the level of GAPDH. Primers were as follows: 5′-ctgctgctttctccctcaac-3′, 5′-gactggcgagccttagtttg-3′ for TGFβ; 5′-tgctgtggctctatggcagg-3′, 5′-ctctgagtgtgatgggagca-3′ for BMP3; 5′-ggctaacagaaccaggacca-3′, 5′-gcccagaagcactagactgg-3′ for activin; and 5′-aactttggcattgtggaagg-3′, 5′-acacattgggggtaggaaca-3′ for GAPDH.

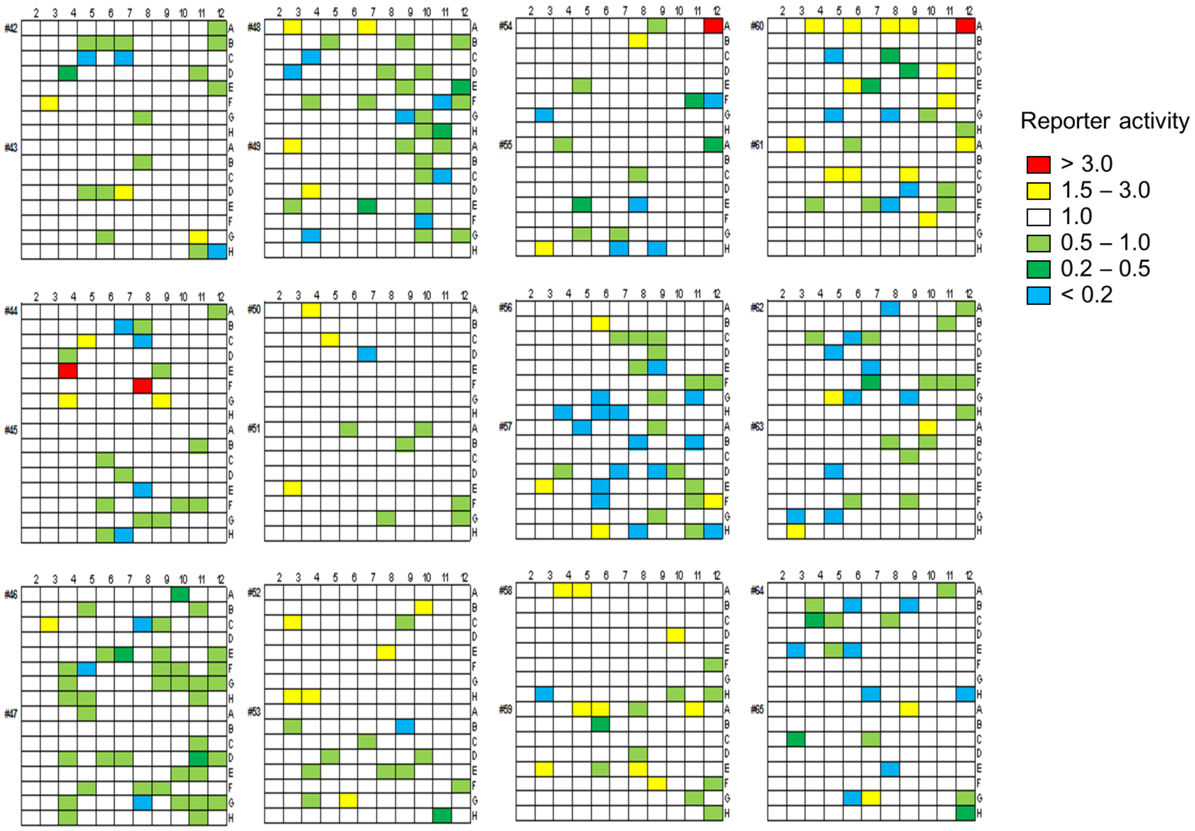

Supplementary Figure S1. **Selection of bioactive chemical compounds on RUNX2-induced promoter activity.** C2C12 cells were transduced with a RUNX2-responsive luciferase gene (6xOSE-luc) and pCMVβ and cultured in 96-well plates. Cells were incubated with chemical compounds (10 μM) for 24 h and subjected to reporter gene assay using a 384-well assay system. Bioactive 384 compounds were selected from 12,259 compounds by the 1.5-fold increase threshold and subjected to the subsequent dose-dependent reporter assay. Ninety eight compounds were selected for the subsequent assays.

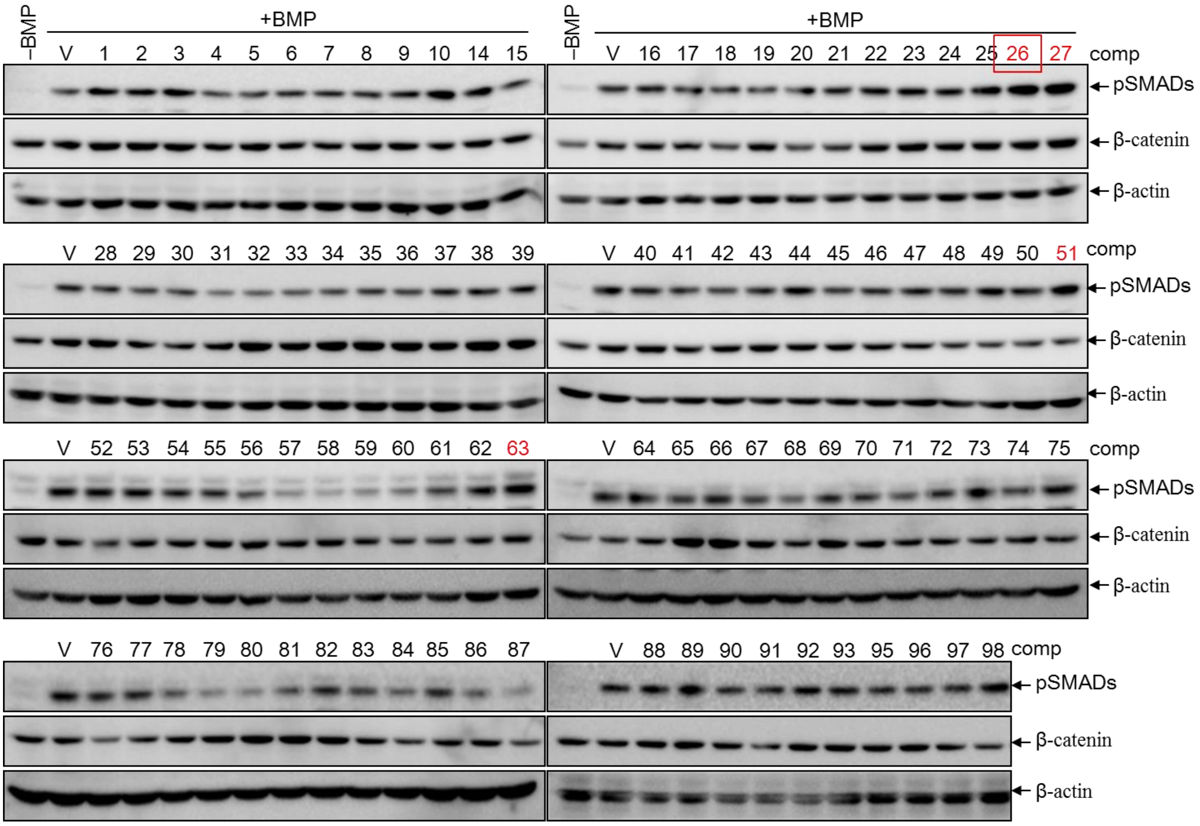

Supplementary Figure S2. **Effects of selected compounds on phosphorylation of SMAD1/5/8 and β-catenin.** C2C12 cells were treated with chemical compounds (10 μM) in the presence of BMP2 (25 ng/ml) for 30 min. Total cell lysates were harvested and resolved by SDS-PAGE, followed by immunoblotting analysis with antibodies against pSMAD1/5/8 and β-catenin.

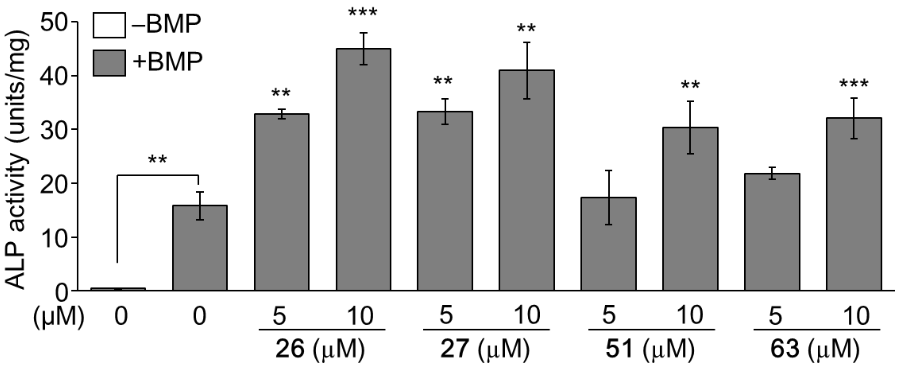

Supplementary Figure S3. Effects of four selected compounds on ALP activity. C2C12 cells were incubated with four selected compounds (**26**, **27**, **51**, and **63**) under osteoblast differentiation conditions that contain BMP2 (25 ng/ml). Total cell extracts were used for ALP activity assay. ALP activity is expressed as units per mg protein. Statistical significance was calculated compared to the activity in BMP2-treated cells. **P < 0.005 and ***P < 0.0005.

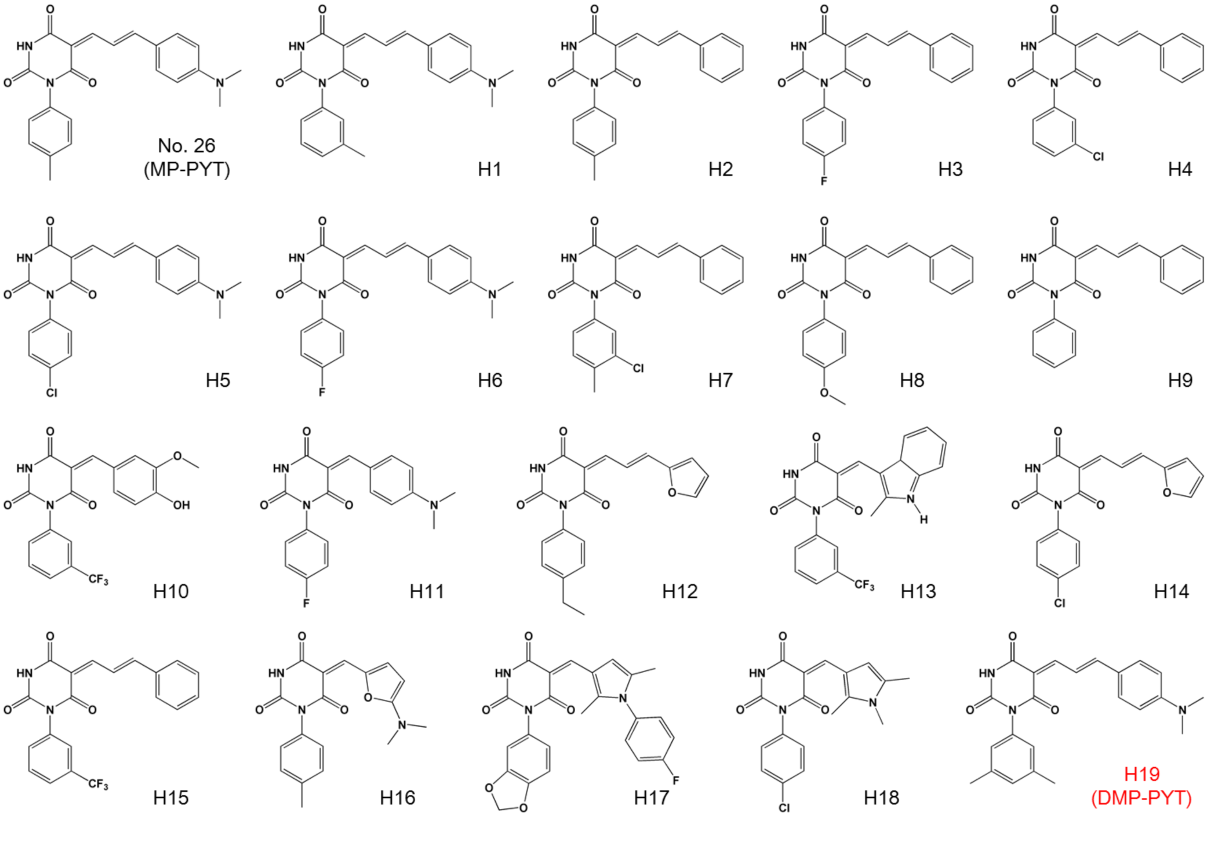

Supplementary Figure S4. Structures of MP-PYT derivatives. Structural derivatives of MP-PYT (Compound **26**) were selected from the chemical library.

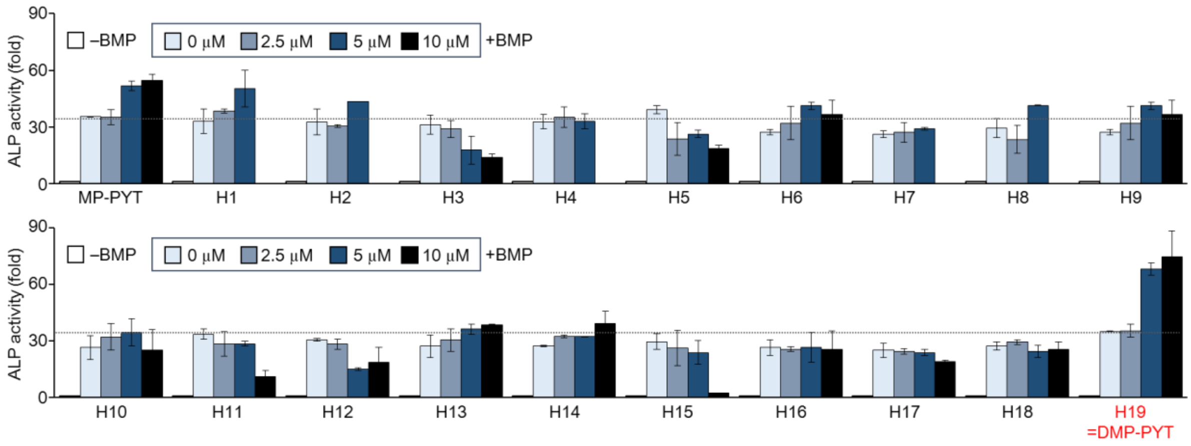

Supplementary Figure S5. Effects of MP-PYT derivatives on ALP activity. C2C12 cells were differentiated into osteoblasts in the presence of BMP2 (25 ng/ml) and also incubated with different amounts of MP-PYT (compound **26**) and its 19 derivatives. After 6 days, cell extracts were used for ALP activity assay. Compound H19 was referred as DMP-PYT.

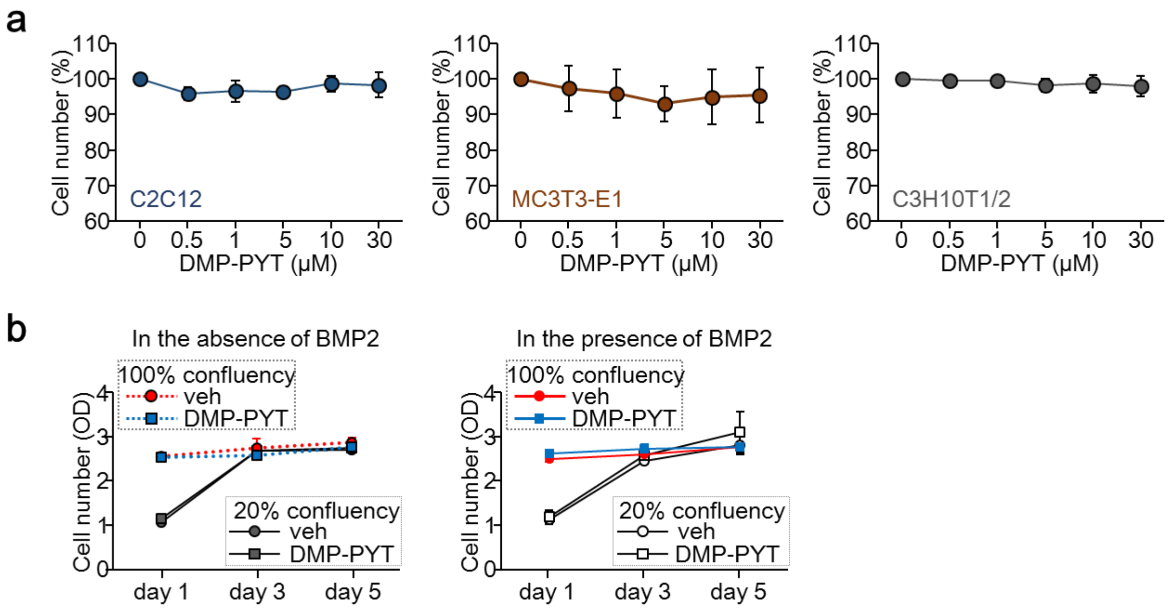

Supplementary Figure S6. No cytotoxic effects of DMP-PYT *in vitro*. (a) C2C12, MC3T3-E1, and C3H10T1/2 cells were incubated with DMP-PYT for 3 days under growing conditions. Cell viability was determined by EZ-Cytotox cell viability assay kit. (b) C2C12 cells were plated with different cell density. 20%- and 100%-confluent C2C12 cells were treated with DMP-PYT (10 μM) in the absence or presence of BMP2 for 5 days. Cell numbers were determined at days 1, 3, and 5 after plating using EZ-Cytotox cell proliferation assay kit.

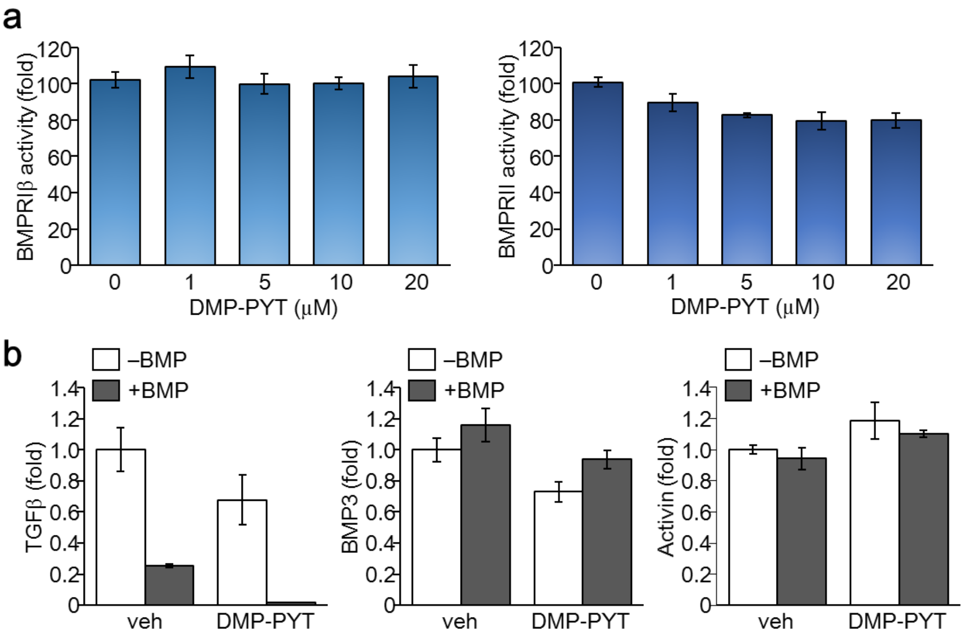

Supplementary Figure S7. No changes in kinase activity of BMPR by DMP-PYT. (a) The effects of DMP-PYT on BMPR1β and BMPRII kinase activity was determined using a radiolabelled substrate (Eurofins Scientific). (b) C2C12 cells were differentiate into osteoblasts in the presence of 10 μM DMP-PYT and/or BMP2 (25 ng/ml) for 6 days. Total RNA was subjected to analysis of the expression of TGFβ, BMP3, and activin.

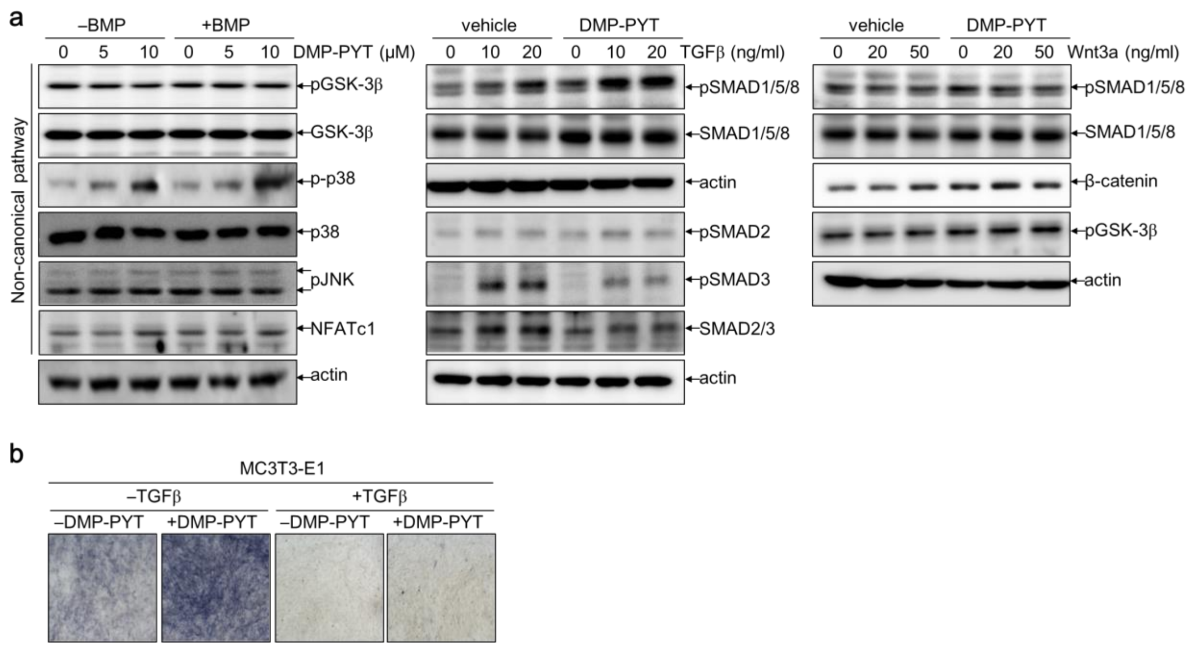

Supplementary Figure S8. Effects of DMP-PYT on non-canonical BMP, TGFβ, and Wnt signaling pathways. (a) C2C12 cells were differentiated into osteoblasts and incubated with DMP-PYT (as indicated concentration or 10 µM) for 30 min in the presence of BMP2 (25 ng/ml), TGFβ (10 and 20 ng/ml), or Wnt3a (20 and 50 ng/ml). Antibodies against pSMAD2, pSMAD3, pSMAD1/5/8, pGSK-3, p-p38, pJNK (Cell signaling), β-catenin (Millipore), p38, NFATc1, SMAD1/5/8, SMAD2/3, and β-actin (Santa Cruz Biotechnology Inc) were used for the immunoblot analysis. (b) MC3T3-E1 cells were treated with either vehicle or DMP-PYT (10 µM) in the absence or presence of TGFβ (10 ng/ml) and induced to differentiate into osteoblasts under differentiation conditions for 10 days. Cells were fixed and stained with ALP.

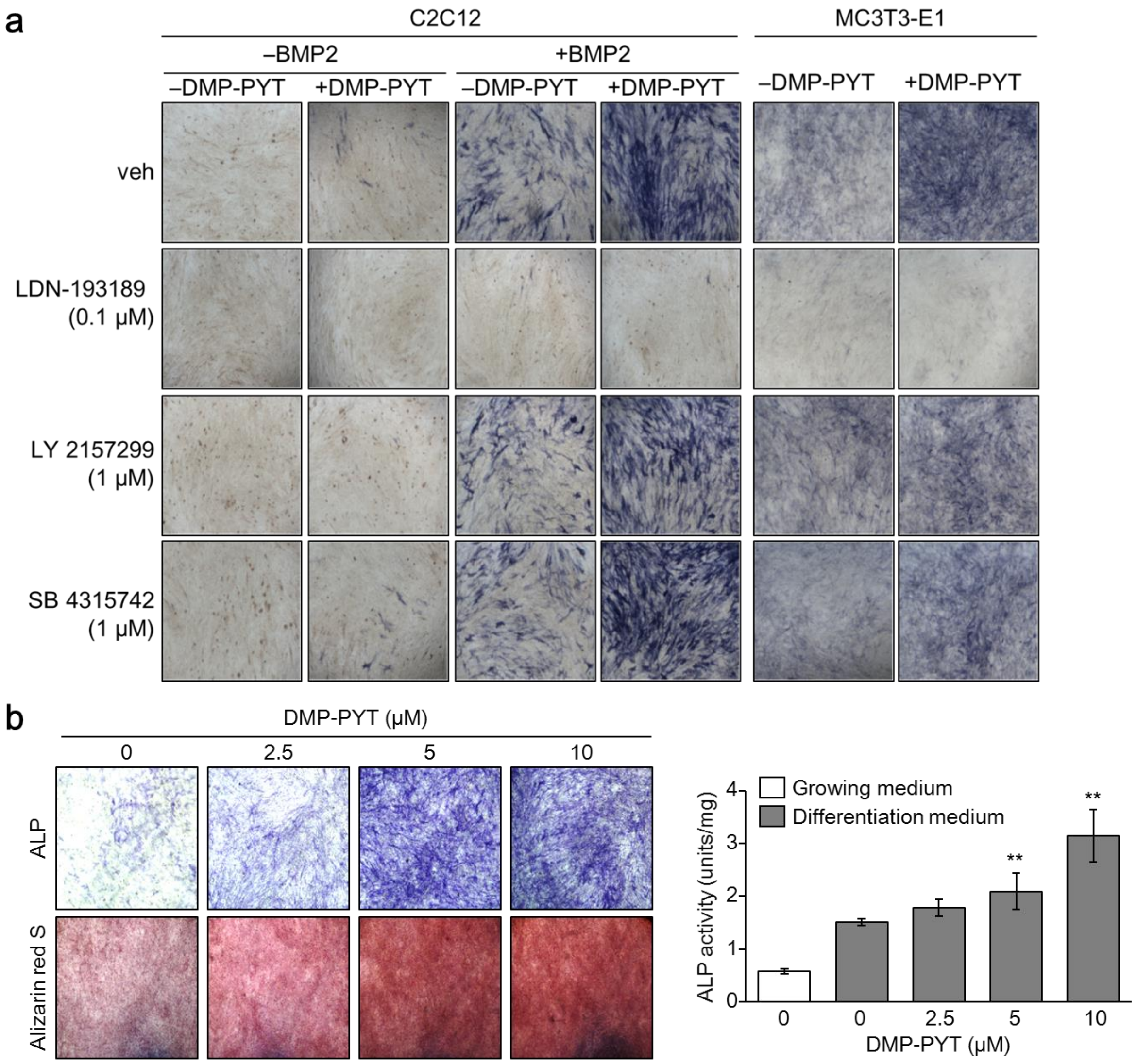

Supplementary Figure S9. Osteogenic activity of DMP-PYT dependent of BMP signaling. (a) C2C12 and MC3T3-E1 cells were differentiated into osteoblasts in the presence of DMP-PYT for 6 days and 10 days, respectively. C2C12 and MC3T3-E1 cells were additionally incubated with BMPR inhibitor (100 nM LDN-193189) or TGFβR inhibitors (1 μM LY2157299 and 1 μM SB4315742) during osteoblast differentiation. Cells were fixed in 10% formalin and stained with ALP. (b) MC3T3-E1 cells were cultured under differentiation conditions containing different concentrations of DMP-PYT for 10 days and subjected to ALP and alizarin red S staining. ALP activity was determined from the cell extracts at day 10. ALP activity is given as units per mg protein of three independent experiments. $^{**}P < 0.005$.

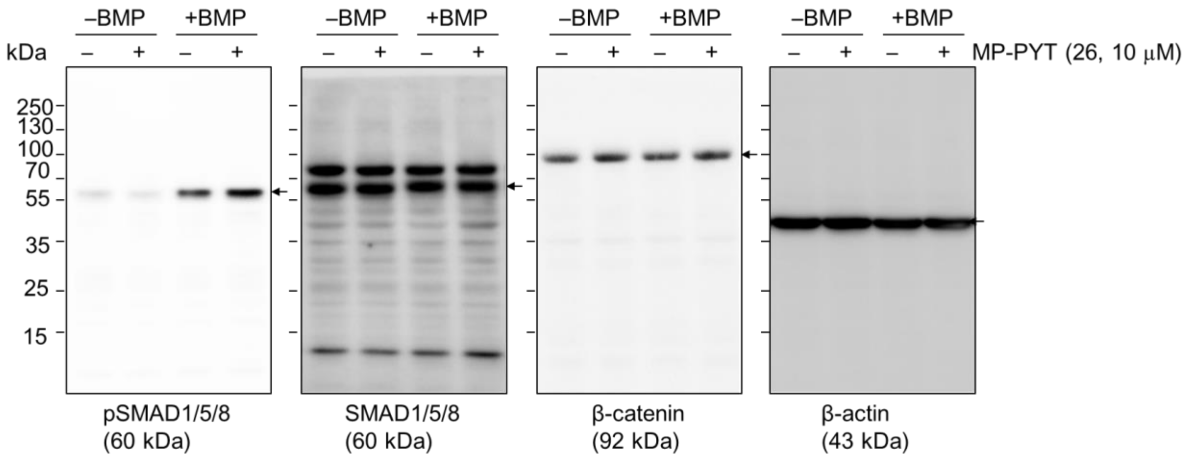

Supplementary Figure S10. Uncropped original images of immunoblot analysis as shown in Figure 1d.

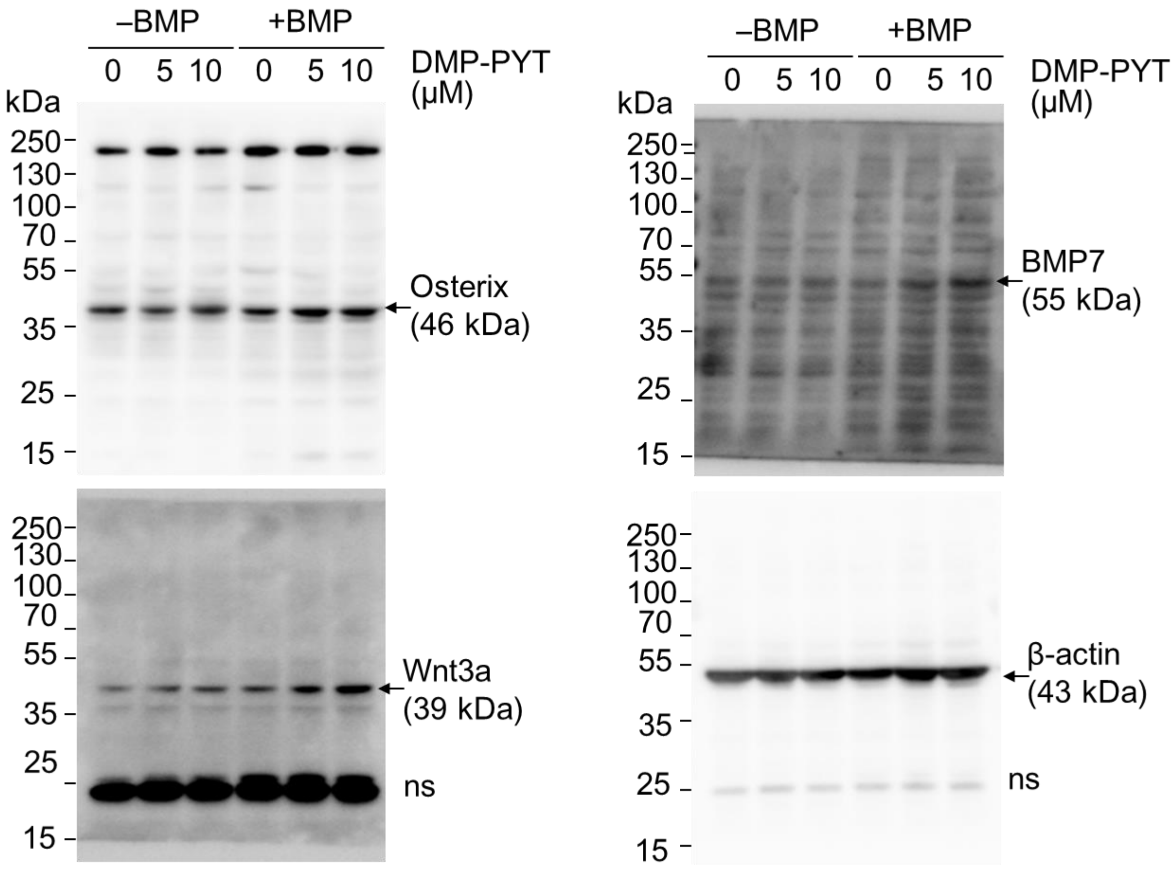

Supplementary Figure S11. Uncropped original images of immunoblot analysis as shown in Figure 3b.

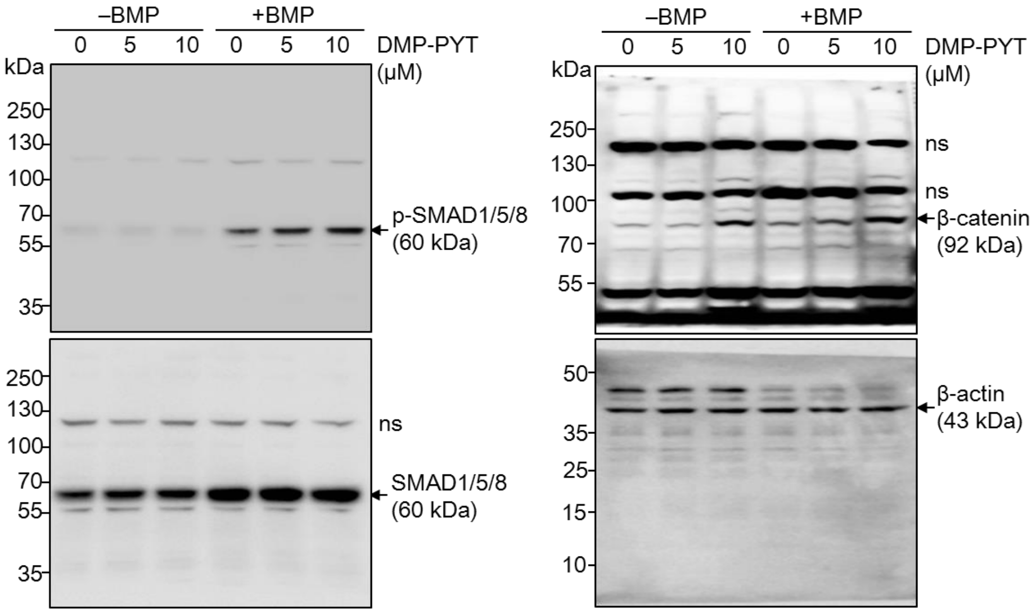

Supplementary Figure S12. Uncropped original images of immunoblot analysis as shown in Figure 4b.

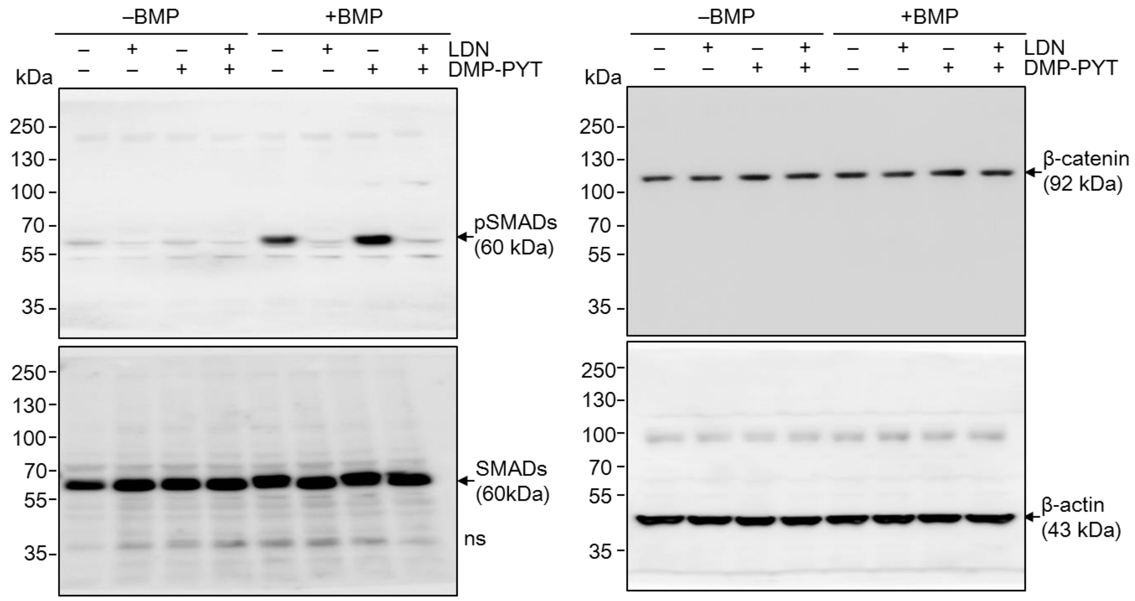

Supplementary Figure S13. Uncropped original images of immunoblot analysis as shown in Figure 4c.

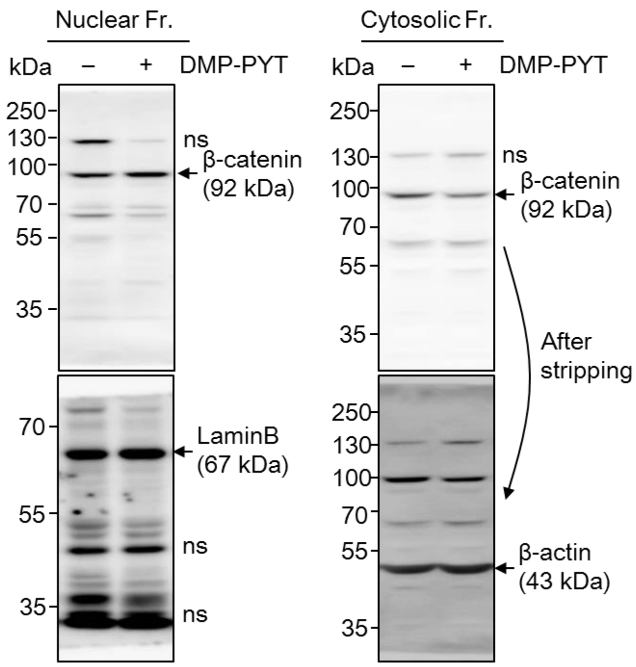

Supplementary Figure S14. Uncropped original images of immunoblot analysis as shown in Figure 5a.

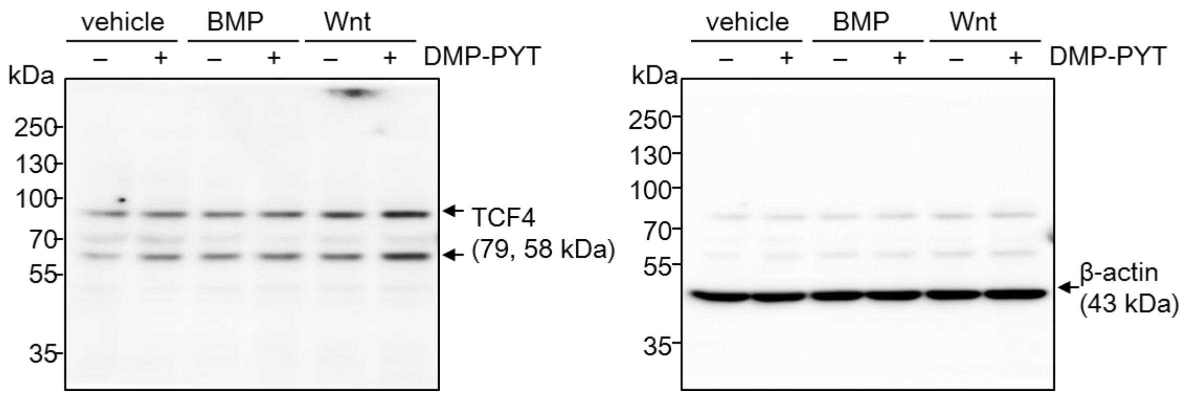

Supplementary Figure S15. Uncropped original images of immunoblot analysis as shown in Figure 5e.

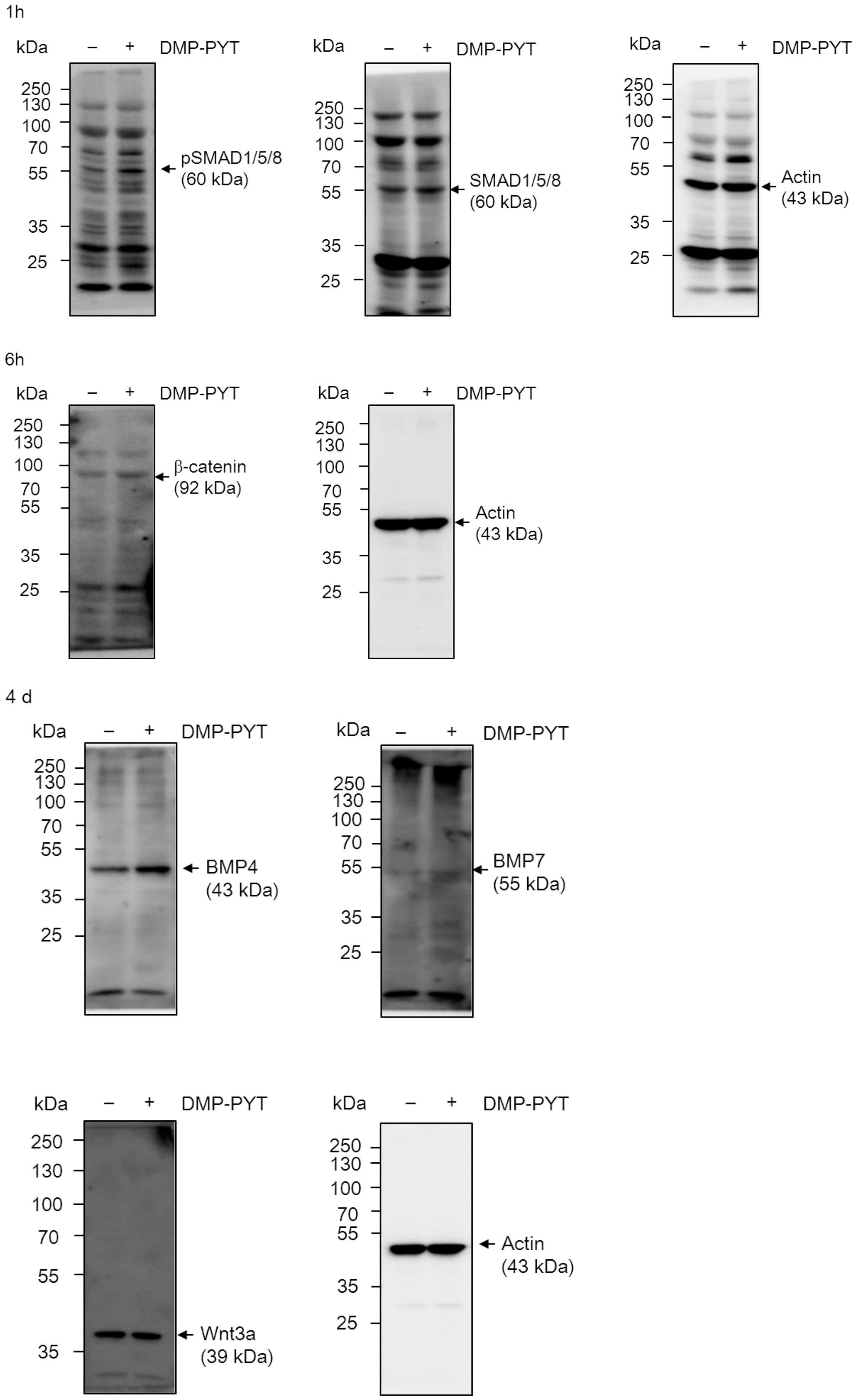

Supplementary Figure S16. Uncropped original images of immunoblot analysis as shown in Figure 6c.

References

1 Kim, H. J. *et al.* Establishment and characterization of a stable cell line to evaluate cellular Runx2 activity. *J Cell Biochem* **91**, 1239-1247 (2004).
